# Comprehensive Analysis of Combinatorial Pharmacological Treatments to Correct Nonsense Mutations in the CFTR Gene

**DOI:** 10.3390/ijms222111972

**Published:** 2021-11-04

**Authors:** Arianna Venturini, Anna Borrelli, Ilaria Musante, Paolo Scudieri, Valeria Capurro, Mario Renda, Nicoletta Pedemonte, Luis J. V. Galietta

**Affiliations:** 1 Telethon Institute of Genetics and Medicine (TIGEM), 80078 Pozzuoli, Italy; a.venturini@tigem.it (A.V.); a.borrelli@tigem.it (A.B.); m.renda@tigem.it (M.R.); 2 U.O.C. Genetica Medica, IRCCS Istituto Giannina Gaslini, 16147 Genova, Italy; ilaria.musante@unige.it (I.M.); paolo.scudieri@unige.it (P.S.); valeriacapurro@yahoo.it (V.C.); nicoletta.pedemonte@unige.it (N.P.); 3 Department of Neurosciences, Rehabilitation, Ophthalmology, Genetics, Maternal and Child Health (DINOGMI), Università degli Studi di Genova, 16132 Genova, Italy; 4 Department of Translational Medical Sciences (DISMET), Università degli Studi di Napoli “Federico II”, 80131 Napoli, Italy

**Keywords:** cystic fibrosis, nonsense mutation, chloride channel, readthrough therapy, CFTR rescue

## Abstract

Cystic fibrosis (CF) is caused by loss of function of the CFTR chloride channel. A substantial number of CF patients carry nonsense mutations in the *CFTR* gene. These patients cannot directly benefit from pharmacological correctors and potentiators that have been developed for other types of CFTR mutations. We evaluated the efficacy of combinations of drugs targeting at various levels the effects of nonsense mutations: SMG1i to protect CFTR mRNA from nonsense-mediated decay (NMD), G418 and ELX-02 for readthrough, VX-809 and VX-445 to promote protein maturation and function, PTI-428 to enhance CFTR protein synthesis. We found that the extent of rescue and sensitivity to the various agents is largely dependent on the type of mutation, with W1282X and R553X being the mutations most and least sensitive to pharmacological treatments, respectively. In particular, W1282X-CFTR was highly responsive to NMD suppression by SMG1i but also required treatment with VX-445 corrector to show function. In contrast, G542X-CFTR required treatment with readthrough agents and VX-809. Importantly, we never found cooperativity between the NMD inhibitor and readthrough compounds. Our results indicate that treatment of CF patients with nonsense mutations requires a precision medicine approach with the design of specific drug combinations for each mutation.

## 1. Introduction

Cystic fibrosis (CF) is a life-threatening autosomal recessive disease that is mainly widespread among Caucasians, with a frequency of 1/3000 newborns [1,2]. CF is caused by mutations in the cystic fibrosis transmembrane regulator (*CFTR*) gene, which encodes for a cAMP-dependent anion channel that mediates chloride and bicarbonate transport at the apical membrane of epithelial cells of airways, pancreas, liver, intestine, and sweat glands [3]. Although CF is a multi-organ disease, the most severe consequences are observed in the lungs, where defective anion secretion alters the chemical and physical properties of the apical fluid that covers the airways [1]. In particular, dehydration, and possibly acidification of airway surface, leads to impairment of mucociliary clearance and antimicrobial mechanisms with the onset and persistence of severe lung infections.

So far, more than 2000 *CFTR* gene variants have been identified, of which 352 variants are confirmed to be CF-causing mutations [2,3]. CF mutations can be grouped into seven classes based on their effects on CFTR maturation, expression, and function [4]. Since the discovery of the *CFTR* gene in 1989, great efforts have been made to identify pharmacological approaches to correct the basic defect in CFTR function. This search has been highly successful, with the development of molecules called CFTR modulators that are essentially divided into potentiators and correctors [2,3]. Potentiators are small molecules that promote the opening of the CFTR channel. They are therefore particularly suited for so called class 3 or gating mutations (e.g., G551D, G1349D, G178R). The most advanced potentiator is VX-770 [5], also known as ivacaftor, which constitutes the active principle of Kalydeco^®^, a drug that has already been approved for the treatment of CF patients carrying at least one of a large list of class 3 and class 4 mutations. CFTR correctors, such as VX-809 [6], are instead small molecules that target the multiple folding, stability, and trafficking defects caused by F508del, the most frequent mutation among CF patients [2,3]. F508del also causes a channel gating defect that requires a combination of VX-770 with correctors to achieve the best effect in rescuing CFTR function [2]. Initial treatments for CF patients carrying the F508del mutation consisted of VX-770 potentiator combined with corrector VX-809 (drug name: Orkambi^®^) or corrector VX-661 (drug name: Symdeko^®^). The most advanced treatment for F508del patients is now represented by Trikafta^®^ (Kaftrio^®^ in Europe), which includes VX-661, VX-770, and VX-445, the last one being a second-generation corrector [7]. VX-809 and VX-661 are compounds with similar mechanism of action, probably involving binding to specific domains of CFTR protein, including the first transmembrane domain (TMD1) and/or the interface between nucleotide-binding domain 1 (NBD1) and TMD2 [8,9,10,11,12]. VX-445 is instead a corrector that is highly effective, particularly if combined with VX-809 or VX-661 [7,13]. Its mechanism of action may involve interaction with the nucleotide-binding domain 1 (NBD1) of CFTR [14]. VX-445 has also been found to act as a CFTR potentiator [15,16]. Importantly, correctors and potentiators are not only effective on the mutations towards which they were originally developed. Actually, they often act on many other CFTR mutants [17].

Despite the success of CFTR pharmacotherapy, there are still 5–10% of CF patients who will not directly benefit from correctors and potentiators because they carry undruggable mutations, such as premature termination codons (PTCs) that lead to a truncated nonfunctional CFTR protein [2,3].

One possible approach for PTCs is to use “readthrough” agents, such as the aminoglycosides (AAGs) gentamycin and G418 [18,19]. These molecules act on the ribosome, promoting the insertion of an amino acid at the position of the nonsense codon, thus allowing full-length protein synthesis [19]. However, gentamycin and G418 cannot be used in vivo because of their severe toxicity. To overcome this problem, chemically modified AAGs with reduced toxicity were generated [20,21]. The most promising compound, NB124 (now known as ELX-02, Eloxx Pharmaceuticals), was effective in a series of cell- and animal-based experiments [19,22] and is presently being tested on CF patients in phase 2 clinical trials [23].

The pharmacological rescue of PTCs implies the design of a combinatorial therapy including other drugs in addition to the readthrough agent. First, the efficacy of readthrough agents is hindered in most cases by the “nonsense-mediated RNA decay” (NMD), a biological mechanism that causes the degradation of most mRNAs carrying a PTC [19,24,25]. This problem can be potentially targeted with small molecules that act as NMD inhibitors [26,27,28,29], such as SMG1i [30]. Second, the readthrough mechanism causes the insertion of an amino acid that is different from the normal one. Therefore, a CFTR protein with reduced stability and gating is frequently generated. These problems can be potentially overcome with the inclusion of correctors and potentiators in the readthrough therapy. Finally, rescue of mutant CFTR can be enhanced by “CFTR amplifiers” [31,32]. These molecules, such as PTI-428, could generally promote CFTR protein synthesis, irrespective of the presence of PTCs or other mutations. Therefore, they are general-purpose drugs in CF. In general, a combinatorial therapy with NMD inhibitors, readthrough agents, and CFTR amplifiers/correctors/potentiators may be envisioned to target PTCs. However, it is important to define which combination is optimal in terms of maximal rescue of CFTR protein and function, and to which extent the rescue is dependent on type and position of the mutation. Indeed, the specific nonsense codon involved and the surrounding nucleotide sequence are both known to affect the efficacy of readthrough agents [19,33]. At the protein level, the position in the CFTR amino acid sequence is also important. An amino acid change, resulting from readthrough, may have different conformational effects depending on the region/domain of CFTR where it occurs.

In our study, we tested several combinations of compounds on a panel of CF-causing PTCs localized at different positions of the *CFTR* coding sequence. Our goal was to identify the minimal combination of pharmacological agents producing the most effective rescue of CFTR function for each mutation. Our results reveal important differences in the sensitivity of the various mutations to rescue maneuvers, with W1282X and R553X as the most sensitive and most refractory mutations, respectively. The identification of the most effective combinatorial treatments for CF PTCs will provide a basis for the planning of future clinical trials.

## 2. Results

For our experiments, we took advantage of the availability of the 16HBE14o- bronchial epithelial cells, provided by the Cystic Fibrosis Foundation, whose *CFTR* gene locus has been edited to introduce various CF-causing PTCs [34]. When seeded on porous membranes, 16HBE14o- cells form electrically tight epithelia that can be conveniently used to study CFTR-dependent Cl^−^ transport. We started our experiments on cells with G542X, which is the most frequent PTC in CF [35]. Epithelia were treated for 24 h with the selected compounds and then mounted in chambers for transepithelial short-circuit current recordings. During recordings, CFTR activity was first stimulated with the membrane-permeable cAMP analog CPT-cAMP, then potentiated with VX-770, and finally blocked with the specific CFTR inhibitor-172 [36]. The amplitude of the current drop caused by the inhibitor was taken as the parameter reflecting CFTR function.

**Figure 1:**
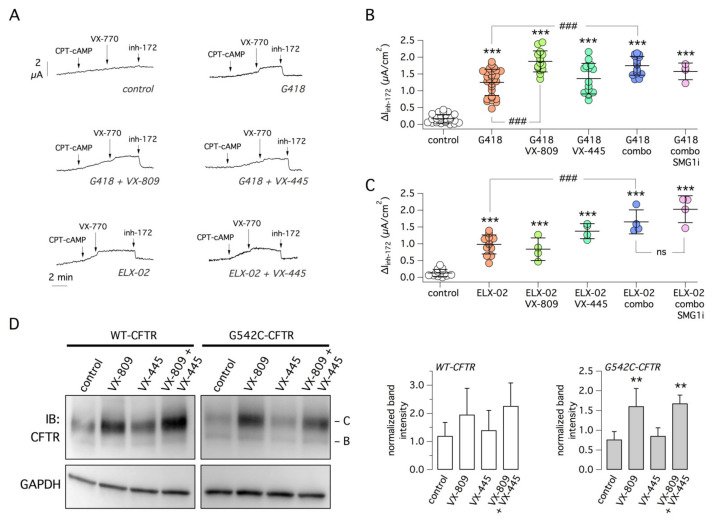
Pharmacological rescue of G542X-CFTR mutant. (**A**) Representative short-circuit current recordings from the 16HBE14o- bronchial epithelial cell line carrying the G542X mutation in the *CFTR* gene. Before recordings, cells were treated for 24 h with G418 (0.5 mg/mL) or ELX-02 (0.5 mg/mL) alone or in combination with VX-809 (1 µM), VX-445 (5 µM), the two correctors together (combo), with/without SMG1i (1 µM). During recordings, cells were first stimulated with the membrane-permeable cAMP analog (CPT-cAMP, 100 µM) and then with VX-770 (1 µM), to induce CFTR activation. CFTR activity was then inhibited with inh-172 (10 µM). Pharmacological rescue results in a higher CFTR activity as indicated by the larger amplitude of inh-172 effect. (**B**) Summary of data obtained with G418 as readthrough agent, with/without correctors and SMG1i. The scatter dot plot reports the amplitude of the current drop elicited by inh-172 for the indicated conditions. ***, *p* < 0.001 vs. control. ###, *p* < 0.001 vs. G418. (**C**) Summary of data obtained with ELX-02 as readthrough agent, with/without other compounds. ***, *p* < 0.001 vs. control; ###, *p* < 0.001 vs. ELX-02; ns, not significant. (**D**) Immunoblot analysis of wild-type and G542C-CFTR protein processing. The image (left) shows a representative Western blot experiment performed on lysates of HEK293 cells transiently transfected with wild-type or G542C-CFTR plasmids and treated for 24 h with VX-809 (1 µM) and/or VX-445 (5 µM). The B and C bands correspond to partially and fully glycosylated forms of CFTR protein. The bar graphs (right) summarize the C band intensity (normalized for GAPDH expression) from Western blot experiments. **, *p* < 0.01 vs. control (*n* = 4).

**Figure 2:**
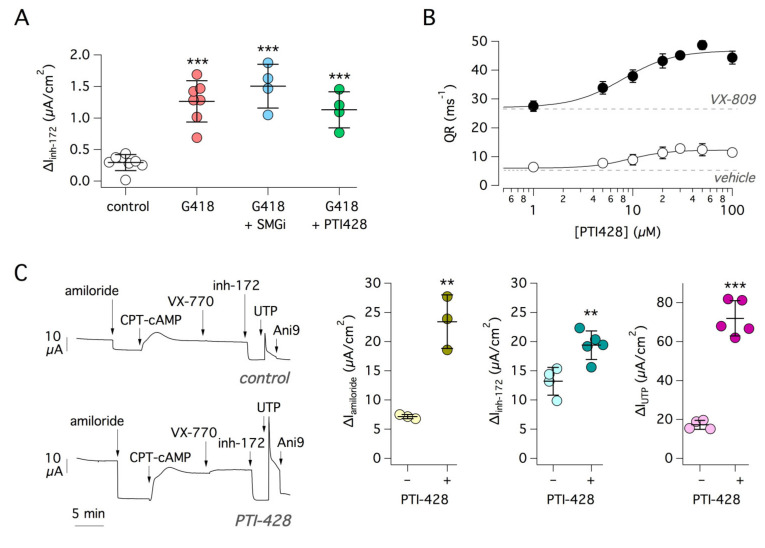
Functional analysis of PTI-428 and SMG1i. (**A**) The scatter dot plot reports CFTR function (i.e., current sensitive to inh-172) in 16HBE14o- cells expressing G542X-CFTR treated for 24 h with G418 (0.5 mg/mL) alone or in combination with SMG1i (1 µM) or PTI-428 (30 µM). SMG1i and PTI-428 appear ineffective under these conditions. ***, *p* < 0.001 vs. control. (**B**) Rescue of F508del-CFTR function by PTI-428. CFBE41o- cells expressing F508del-CFTR were treated for 24 h with the indicated PTI-428 concentrations, in the presence of absence of VX-809 (1 µM). Data (quenching rate, QR), obtained with the HS-YFP assay, were fitted with a Hill function. Dashed lines indicate the activity with VX-809 or vehicle alone. (**C**) Representative traces (left) show Ussing chamber experiments done on non-CF primary bronchial epithelial cells. Cells were treated for 24 h with the CFTR amplifier PTI-428 (30 µM) or vehicle (DMSO) alone. During recordings, the function of three channels was evaluated: (i) for the epithelial Na^+^ channel (ENaC), we measured the amplitude of amiloride (10 µM) effect; (ii) for CFTR function, we measured the effect of inh-172 after maximal stimulation with CPT-cAMP; (iii) for the activity of the Ca^2+^-activated Cl^−^ channel TMEM16A, we measured the maximal amplitude of the current elicited with UTP (100 µM). Ani9 (5 µM) was added to inhibit TMEM16A at the end of the experiments. The scatter dot plot (right) reports the function of ENaC, CFTR, and TMEM16A in cells treated with PTI-428 or vehicle. **, *p* < 0.01; ***, *p* < 0.001 vs. control.

As shown in Figure 1, G542X cells under control conditions showed near negligible response to CFTR stimulation and inhibition. However, treatment with the readthrough agents G418 or ELX-02 (at 0.5 mg/mL, i.e., 722 and 736 µM, respectively, the optimal concentration based on preliminary experiments) significantly enhanced function (~9-fold and 7-fold, respectively; Figure 1A–C). The effect of G418 was further enhanced by the combination with corrector VX-809 but not with VX-445. The rescue by ELX-02 was instead improved by the combination of VX-809 and VX-445 (Figure 1C). Importantly, the addition of the NMD inhibitor SMG1i to the triple combinations G418/VX-809/VX-445 or ELX-02/VX-809/VX-445 did not further increase G542X-CFTR function (Figure 1B,C). SMG1i was also tested with G418, without correctors (Figure 2A), but did not show an additive effect.

To investigate the effect of VX-809 and VX-445 at the protein level, we tested both compounds on the expression of G542C-CFTR, the CFTR variant that is considered the most favored outcome of G542X readthrough [33]. HEK293 cells were transfected with the G542C-CFTR expression vector, treated with or without correctors, lysed, and CFTR protein analyzed by immunoblot. Treatment with VX-809, but not with VX-445, significantly enhanced the expression of G542C-CFTR (Figure 1D). For comparison, we carried out similar experiments with wild-type CFTR. Treatment with correctors did not significantly change CFTR expression (Figure 1D).

VX-809 can be potentially replaced by VX-661, as they both share a similar mechanism of action. Therefore, we tested VX-661 in combination with G418 and compared the results with those obtained with VX-809 plus G418 (Figure S1). In contrast with VX-809, VX-661 did not significantly improve the rescue by G418. This result can be explained with a reduced corrector efficacy of VX-661 compared with VX-809 [13].

We explored the possibility of improving the rescue of G542X-CFTR using PTI-428, a CFTR amplifier [31,32]. PTI-428 did not increase CFTR function above the level achieved with G148 alone (Figure 2A). The lack of effect of the amplifier was surprising since this compound has been proposed as a general-purpose modulator, similarly effective on wild-type and mutant CFTR because it promotes enhanced CFTR protein expression by stabilization of the CFTR transcript [32]. Accordingly, we evaluated the effect of PTI-428 by real-time RT-PCR. PTI-428 did not increase CFTR mRNA (Figure S2). A modest increase was observed when PTI-428 was combined with G418.

We tested PTI-428 on CFBE41o- cells expressing F508del-CFTR, whose activity was assessed with the halide-sensitive yellow fluorescent protein (HS-YFP) assay [37,38]. PTI-428 was tested at different concentrations, alone or in combination with VX-809. Incubation with PTI-428 for 24 h increased F508del-CFTR function in a dose-dependent manner (Figure 2B). Combination of PTI-428 with VX-809 resulted in a synergistic effect since the maximal activity of the amplifier in the presence of CFTR corrector was nearly 3 times larger than that measured in its absence (Figure 2B).

We further verified PTI-428 on non-CF bronchial epithelial cells. We expected a change in the current activated by cAMP that was due to CFTR-dependent Cl^−^ secretion. Indeed, treatment with PTI-428 (30 µM, 24 h) increased CFTR function, as shown by the larger amplitude of the block caused by inh-172 (Figure 2C). Intriguingly, the CFTR amplifier also had a significant effect on other electrogenic mechanisms. The effect of amiloride, which is a reporter of the activity of the epithelial Na^+^ channel ENaC, was augmented by the treatment with PTI-428 (Figure 2C). Furthermore, the amplifier also markedly increased the response to UTP, a purinergic agonist that, by mobilizing intracellular Ca^2+^, activates the TMEM16A (ANO1) Cl^−^ channel (Figure 2C).

**Figure 3:**
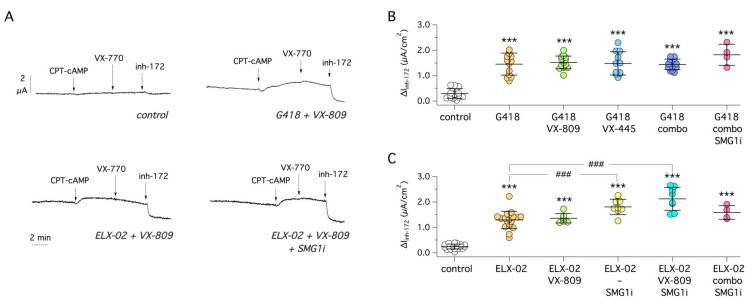
Pharmacological rescue of Y122X-CFTR mutant. (**A**) Representative short-circuit current traces from 16HBE14o- cells expressing Y122X-CFTR treated with the indicated compounds. (**B**) Scatter dot plot summarizing functional results obtained with combinations of G418, VX-809 (1 µM), VX-445 (5 µM), VX-809 plus VX-445 (combo), and SMG1i (1 µM). ***, *p* < 0.001 vs. control. (**C**) Scatter dot plot summarizing functional results obtained with ELX-02 as readthrough agent, with/without correctors and/or SMG1i. ***, *p* < 0.001 vs. control; ###, *p* < 0.001 vs. ELX-02.

After G542X, we studied Y122X, which affects a residue in TMD1. Under control conditions, CFTR activity was nearly negligible. Both readthrough agents, G418 and ELX-02, induced a significant ~ 5-fold increase in CFTR currents, as indicated by the appearance of a current sensitive to CFTR inhibitor (Figure 3A–C). Inclusion of correctors or SMG1i did not appear to improve the effect of G418, while a modest but significant effect was observed by including SMG1i in the treatments together with ELX-02 alone or in combination with VX-809 (Figure 3C). It is interesting to note that with EXL-02 treatments, CFTR appeared to be active under basal conditions, before stimulation with the cAMP agonist. This is a behavior similar to that of wild-type CFTR (see Figure 10A).

**Figure 4:**
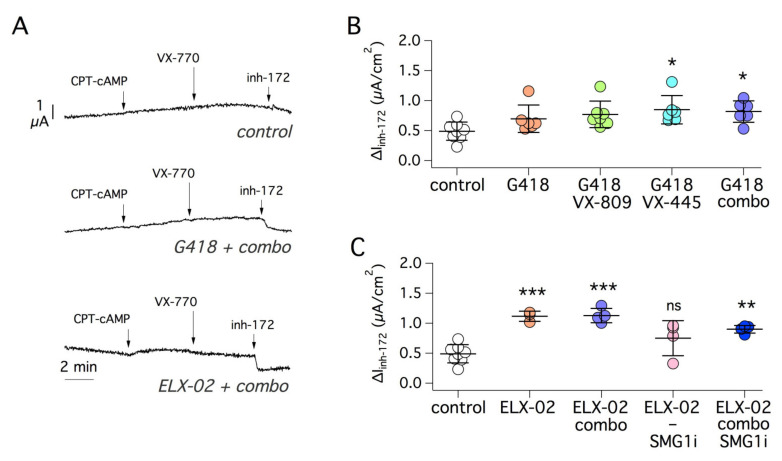
Pharmacological rescue of R553X-CFTR mutant. (**A**) Representative short-circuit current traces from experiments on 16HBE14o- cells expressing R553X-CFTR. (**B**,**C**) Summary of functional data obtained after treating the cells for 24 h with different combinations of readthrough agents (G418 or ELX-02, 0.5 mg/mL), VX-809 (1 µM), VX-445 (5 µM), VX-809 plus VX-445 (combo), and SMG1i (1 µM). *, *p* < 0.05 vs. control; **, *p* < 0.01 vs. control; ***, *p* < 0.001 vs. control; ns, not significant.

We continued our tests on two other PTCs, R553X and R1162X. The former mutation, which resides in NBD1, appeared relatively insensitive to rescue. G418 alone was ineffective but a very modest effect was obtained by combining the readthrough agent with correctors (Figure 4A,B). On the contrary, a small but significant CFTR rescue was observed with ELX-02 alone (Figure 4B). No further rescue was detected by including correctors or SMG1i (Figure 4A,C).

**Figure 5:**
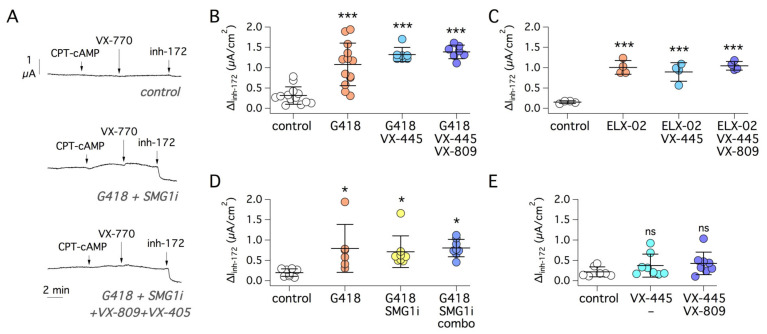
Pharmacological rescue of R1162X-CFTR mutant. (**A**) Representative short-circuit current traces from experiments on 16HBE14o- cells expressing R1162X-CFTR. (**B**,**C**) Scatter dot plots summarizing the effect of G418 or ELX-02 (both at 0.5 mg/mL) with/without correctors VX-809 (1 µM) and VX-445 (5 µM). ***, *p* < 0.001 vs. control. (**D**) Data obtained with G418, with/without SMG1i (1 µM), and VX-809/VX-445 (combo). *, *p* < 0.01 vs. control. (**E**) Data obtained with CFTR correctors, VX-809 (1 µM) and VX-445 (5 µM), in the absence of readthrough agents or NMD inhibitor. ns, not significant.

Regarding R1162X, localized in the linker region between the second transmembrane domain (TMD2) and the second nucleotide binding domain (NBD2), we found rescue by both G418 and ELX-02 agents but no further increase by co-treatment with correctors (Figure 5A–C). As also seen for Y122X, G542X, and R553X, inclusion of NMD inhibitor SMG1i did not enhance the efficacy of the readthrough agent (Figure 5D). We also asked whether the truncated CFTR protein resulting from the R1162X mutation, which allows synthesis of a relatively large portion of CFTR (1162 out of 1480 total amino acids), can be rescued by correctors alone. However, we found no significant response to VX-809 and VX-445 (Figure 5E).

**Figure 6:**
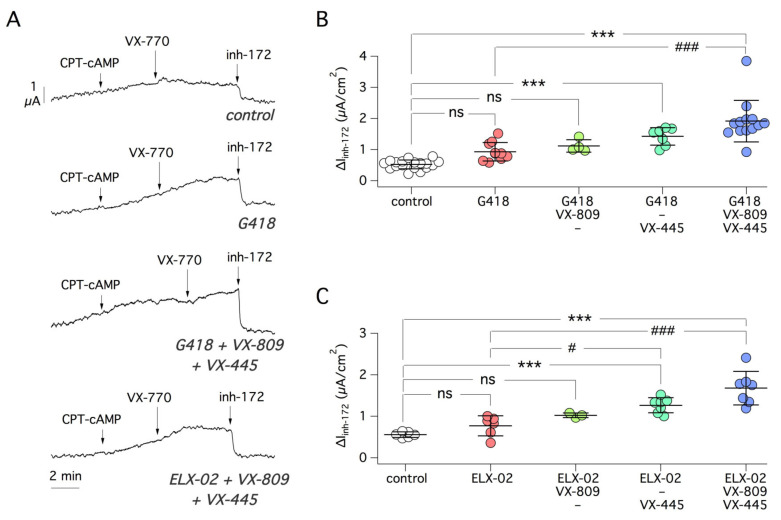
Evaluation of readthrough agents on W1282X-CFTR. (**A**) Representative short-circuit current traces from 16HBE14o- cells expressing W1282X-CFTR, treated for 24 h with the indicated compounds. (**B**,**C**) Summary of data reporting CFTR function (amplitude of inh-172 effect). Cells were treated with G418 0.5 mg/mL or ELX-02 0.5 mg/mL, alone or in combination with CFTR correctors VX-809 (1 µM) and/or VX-445 (5 µM). ***, *p* < 0.001 vs. control. #, *p* < 0.05; ###, *p* < 0.001 vs. G418 or ELX-02.

We next studied 16HBE14o- cells with the W1282X mutation, which is localized in NBD2 and is the second most frequent nonsense mutation in CF [35]. Importantly, activity of W1282X-CFTR was not increased by G418 and ELX-02 but required a combination of the readthrough agents with the VX-809/VX-445 (Figure 6A–C). Actually, VX-445 appeared to contribute more to the rescue with respect to VX-809.

**Figure 7:**
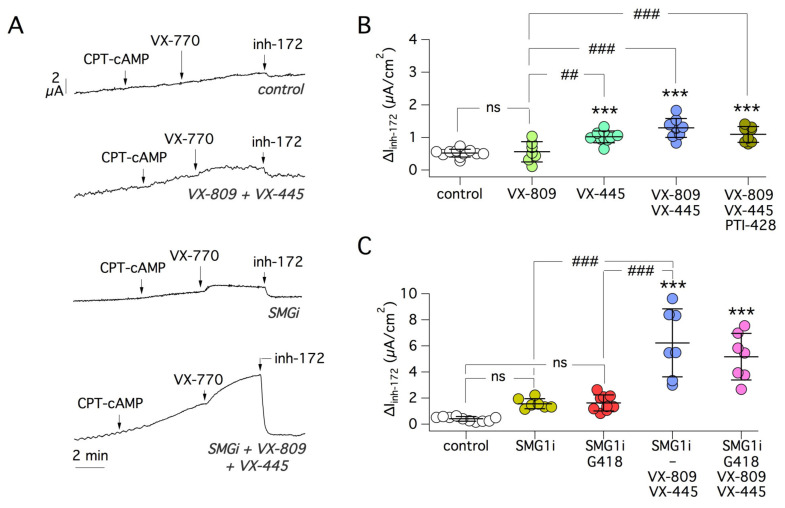
Sensitivity of W1282X-CFTR to correctors and SMG1i. (**A**) Representative short-circuit current traces from 16HBE14o- cells expressing W1282X-CFTR. Cells were treated for 24 h with indicated compounds. (**B**) Scatter dot plot summarizing the results obtained after treatments with CFTR correctors (VX-445, 5 µm; VX-809, 1 µM) and PTI-428 (30 µM). ***, *p* < 0.001 vs. control. ##, *p* < 0.01; ###, *p* < 0.001 vs. VX-809. (**C**) Scatter dot plot summarizing the results obtained with SMG1i (1 µM) with/without the indicated combinations of compounds (VX-445, 5 µM; VX-809, 1 µM; G418, 0.5 mg/mL). ***, *p* < 0.001 vs. control. ###, *p* < 0.001 vs. SMG1i or SMG1i plus G418.

On the basis of the same rationale applied for R1162X, we also tested correctors in the absence of readthrough agent. In contrast with R1162X, we found that W1282X can be significantly rescued with correctors, particularly by VX-445 (Figure 7A,B). Similar to G542X, we found no additional rescue by including PTI-428 in the treatments (Figure 7B). It is interesting to notice that the extent of rescue obtained with correctors alone was comparable with that observed when G148 or ELX-02 were also included (compare Figure 6B,C and Figure 7B). These findings indicate that the rescue is essentially due to correctors and that readthrough agents are ineffective on W1282X. We also tested the effect of SMG1i. In contrast with all other PTCs, functional rescue of W1282X-CFTR appeared markedly sensitive to the NMD inhibitor. In particular, a strong effect was observed when SMG1i was combined with CFTR correctors (Figure 7A,C). A response to VX-770 potentiator was also clearly visible. The total rescue effect was equal to a 17-fold increase relative to vehicle-treated cells. We included G418 in the treatments to evaluate the possibility of a cooperative effect between readthrough and NMD suppression. However, we found no improvement when G418 was included in SMG1i-based treatments (Figure 7C).

**Figure 8:**
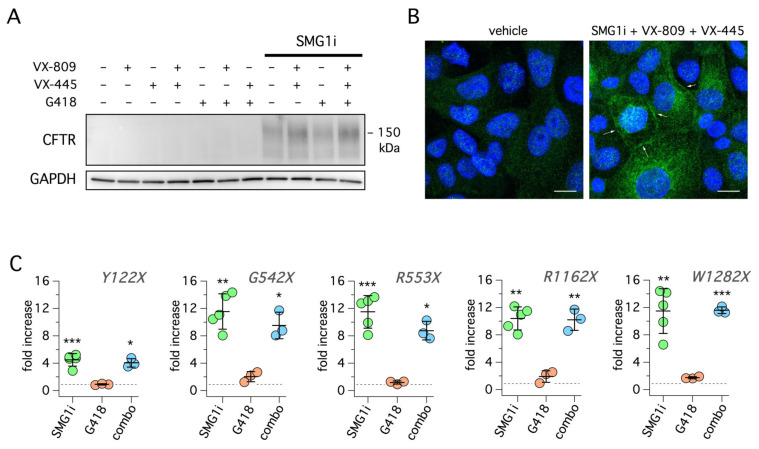
Molecular analysis of SMG1i effects. (**A**) Representative Western blot experiment performed on 16HBE14o- cells expressing W1282X-CFTR. Cells were treated for 24 h with the different combinations of indicated compounds. Treatment with SMG1i (1 µM) was required to evoke the appearance of CFTR protein. The apparent molecular size of the rescued protein is smaller than wild-type CFTR, in agreement with the presence of the truncating mutation. (**B**) Representative confocal microscopy images obtained from 16HBE14o- cells expressing W1282X-CFTR mutation under control condition or after treatment with SMG1i (1 µM) + VX-809 (1 µM) + VX-445 (5 µM). CFTR expression and localization was detected by staining with a specific antibody (green signal). Treatment causes the appearance of CFTR signal in the cytosol and at the plasma membrane (arrows). Nuclei (DAPI staining) are shown in blue. Scale bar: 10 µm. (**C**) Results of real-time RT-PCR experiments to evaluate CFTR mRNA levels in 16HBE14o- cells with the indicated mutations. Before RNA extraction, cells were treated for 24 h with G418 (0.5 mg/mL), SMG1i (1 µM), or with the combination of the two compounds (combo). The scatter dot plots show CFTR transcript fold-change relative to control condition for every mutation. All values were normalized to B2M. The *p* value was determined using one-sample t-test. *, *p* < 0.05; **, *p* < 0.01; ***, *p* < 0.001 vs. control (dashed line).

Given the large effect obtained by SMG1i on W1282X-CFTR function, we carried out further studies to understand the underlying molecular basis. First, we evaluated the effect of SMG1i on W1282X-CFTR protein by immunoblot (Figure 8A). We were not able to detect the protein in lysates of cells treated with G148 in the absence/presence of correctors. Instead, a CFTR signal appeared in lysates of cells treated with SMG1i in agreement with functional data (Figure 8A). Importantly, we found no evidence of a shift to molecular weight when G418 was included with SMG1i, thus confirming the lack of readthrough efficacy for this mutation.

Second, we evaluated the rescue of W1282X-CFTR protein by immunofluorescence (Figure 8B). Treatment of W1282X cells with SMG1i plus correctors caused the appearance of CFTR immunoreactivity, with part of the signal visible at the cell periphery, consistent with plasma membrane localization. Importantly, the localization at the cell periphery was also visible with SMG1i alone, without correctors (Figure S3). In contrast, no signal was found for G542X cells treated with G418 plus correctors (Figure S3), which indicates that the rescue of G542X-CFTR was below the level of detection with this technique. Third, we investigated the extent of CFTR mRNA changes in cells with W1282X compared with the other mutations. Real-time RT-PCR revealed a very large increase in mRNA levels by SMG1i for all mutations except for Y122X (Figure 8C). For this mutation, the increase was approximately 5-fold whereas for all other mutations the increase was more than 10-fold. The quantitation of mRNA was also performed on cells treated with G418 alone or with G418 plus SMG1i. The readthrough agent did not enhance mRNA levels by itself, nor did it appreciably alter the effect of SMG1i (Figure 8C).

**Figure 9:**
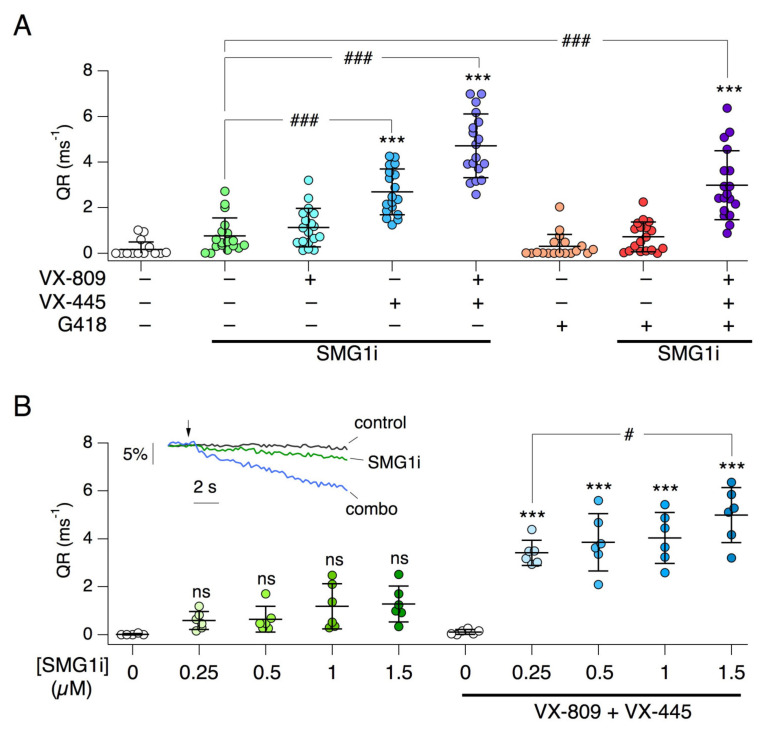
Functional analysis of W1282X-CFTR rescue with the HS-YFP assay. (**A**) Scatter dot plot reporting W1282X-CFTR activity (quenching rate, QR) in cells treated with the indicated combinations of compounds: SMG1i (1 µM), VX-809 (1 µM), VX-445 (5 µM), G418 (0.5 mg/mL). ***, *p* < 0.001 vs. control. ###, *p* < 0.001 vs. SMG1i alone. (**B**) Dose–response relationship for SMG1i in the absence and in the presence of CFTR correctors. ***, *p* < 0.001 vs. no SMG1i. #, *p* < 0.05 between indicated conditions. The inset shows representative traces from the HS-YFP assay in which iodide-rich solution is added (arrow) to start fluorescence quenching caused by iodide influx through CFTR. Conditions were vehicle alone (control), 1 µM SMG1i, and the combination of SMG1i plus VX-809 and VX-445 (combo).

Since W1282X-CFTR shows a large response to rescue treatments, we asked whether it was possible to detect this effect with the HS-YFP assay. W1282X cells were transduced with the HS-YFP coding sequence to obtain stable expression. Cells were then treated for 24 h with different treatments, and CFTR function was then determined by calculating the HS-YFP quenching rate [37,38]. In agreement with short-circuit recordings, we found a significant rescue in cells treated with SMG1i plus correctors (Figure 9A). In particular, VX-445, and not VX-809, was essentially responsible for the functional rescue of W1282X-CFTR in cells treated with SMG1i. We also confirmed that inclusion of G418 did not improve the effect of SMG1i plus correctors. Actually, there was a trend towards a decrease in rescue, which may suggest that the readthrough agent and the NMD inhibitor interfered with each other (see Section 3). We also tested SMG1i at different concentrations, with and without correctors. In the presence of correctors, a SMG1i concentration as low as 0.25 µM was still effective (Figure 9B). The availability of the HS-YFP assay for W1282X-CFTR allowed us to rapidly test a variety of other conditions. First, we tested the quadruple combination consisting of ELX-02, VX-809, VX-445, and SMG1i that could not be studied in short-circuit current experiments. The results (Figure S4A) revealed no improvement with respect to the triple combination VX-809/VX-445/SMG1i, thus confirming the inability of readthrough agents (G418 or ELX-02) to cooperate with SMG1i in the rescue of W1282X-CFTR. We also tested amlexanox, as a second type of NMD inhibitor. This compound was ineffective (Figure S4B).

Finally, we used the HS-YFP assay to test the possibility that VX-445 acts as a potentiator on W1282X-CFTR. Replacing VX-770 with VX-445 in the activating cocktail including CPT-cAMP did not result in significant W1282X-CFTR activation (Figure S4C).

**Figure 10:**
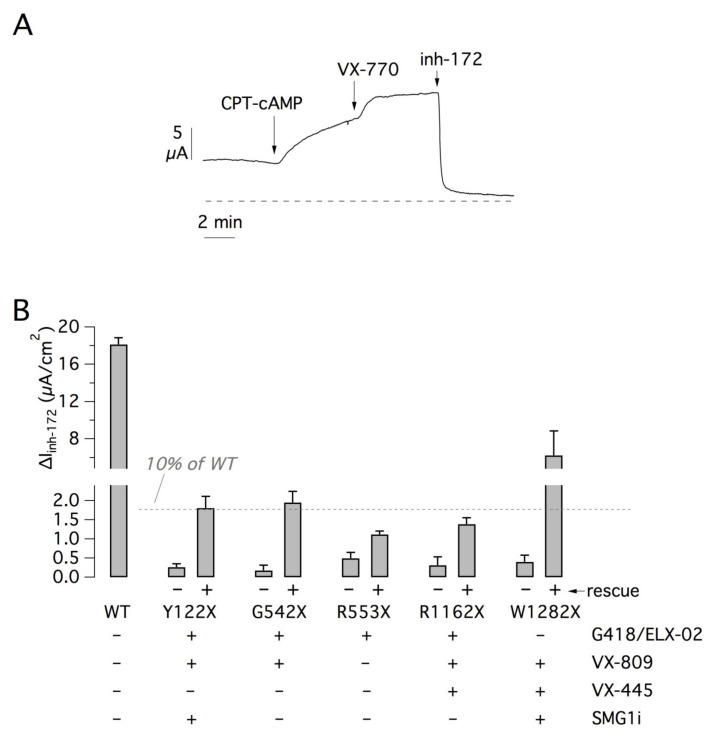
Extent of mutant CFTR rescue by pharmacological treatment. (**A**) Representative short-circuit current recording from 16HBE14o- cells expressing wild-type CFTR. (**B**) Bar graph showing the comparison of CFTR function in 16HBE14o- cells expressing wild-type or mutant CFTR. For cells carrying mutant CFTR, the bars report CFTR function (mean plus SD) under control conditions and after rescue with the most effective treatment for each mutation as indicated.

We carried out transepithelial current recordings on parental 16HBE14o- cells expressing wild-type CFTR and compared the results with those obtained in mutant CFTR cells, under control conditions and after the most effective treatment for each mutation (Figure 10A,B). The average amplitude of CFTR current in the parental cell line was nearly 18 µA. R553X and R1162X were the least responsive mutations. After treatment, Y122X and G542X showed CFTR currents that were close to 10% of normal CFTR function. W1282X was the most responsive mutation since treated cells showed 34% of normal CFTR function (Figure 10B).

**Figure 11:**
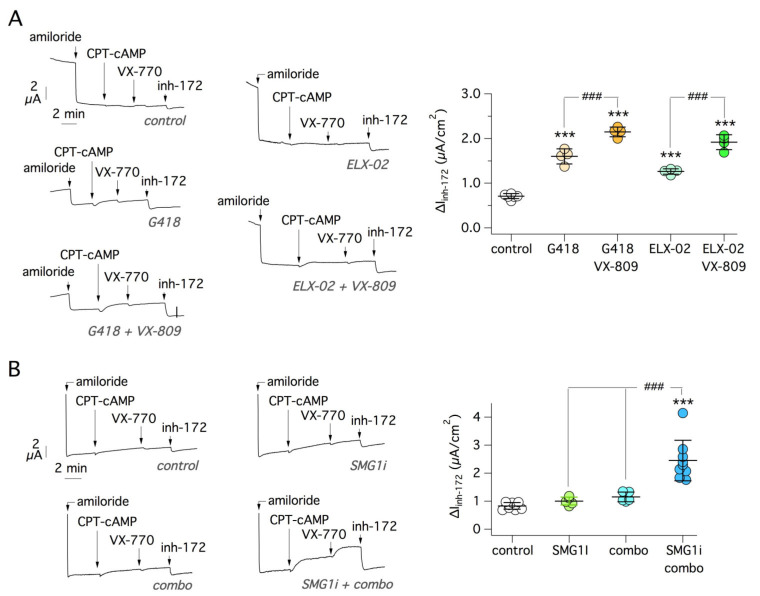
Evaluation of mutant CFTR rescue in native bronchial epithelial cells. (**A**) Short-circuit current traces (left) and summary of data (right) obtained from bronchial epithelial cells of a G542X/1717-1G > A patient. Epithelia were treated with the indicated compounds (0.5 mg/mL G418 and ELX-02, 1 µM VX-809). ***, *p* < 0.001 vs. control. ###, *p* < 0.001 vs. readthrough agent alone. (**B**) Short-circuit current traces (left) and summary of data (right) obtained from bronchial epithelial cells of a W1282X/W1282X patient. Epithelia were treated with the indicated compounds (1 µM SMG1i; 1 µM VX-809; 5 µM VX-445). The amiloride-sensitive currents in the traces were left out of scale to allow a better vision of CFTR currents. ***, *p* < 0.001 vs. control. ###, *p* < 0.001 vs. indicated treatments.

To further validate our results, we carried out experiments on native bronchial epithelial cells from CF patients with nonsense mutations. First, we studied cells from a patient carrying the G542X and 1717-1G > A mutations. The second mutation affects a canonical splice site leading to full disruption of CF protein synthesis [39]. It is therefore an unrescuable mutation [40]. Epithelia generated from the cells of this patient were studied with short-circuit current recordings. In agreement with data obtained in 16HBE14o- cells carrying the G542X mutation, we found a significant rescue following treatment with G418 or ELX-02 (Figure 11A). The effect of the readthrough agents was enhanced by VX-809 (Figure 11A).

In a second set of experiments, we tested epithelia generated from bronchial epithelial cells of a W1282X/W1282X patient. These experiments confirmed the results obtained in gene-edited 16HBE14o- cells: a significant recovery of CFTR function was detected by treatment with SMG1i plus VX-809/VX-445 correctors (Figure 11B). It is interesting to notice that the mean value of CFTR current recorded in bronchial epithelia with G542X and W1282X mutations after most effective treatments was 1.9 and 2.5 µA/cm^2^, respectively. Compared with CFTR currents recorded in non-CF epithelia (~13 µA/cm^2^; Figure 2C), such values correspond to ~15% and 19% of normal CFTR function.

## 3. Discussion

Our study represents a comprehensive analysis of PTC rescue by different pharmacological agents acting at different levels of CFTR protein biosynthesis and processing. There have been already articles reporting the effect of some of the pharmacological agents tested in our experiments [33,34,41,42,43,44]. However, our study includes multiple agents and PTCs within a common framework of assays and cell models. Importantly, we took advantage of a bronchial epithelial cell line in which the endogenous *CFTR* locus was edited to introduce the various nonsense mutations [34]. Using isogenic cell lines is advantageous for minimizing confounding factors due to different genetic background. Furthermore, study of PTC rescue requires that the mutation be present in the gene (instead of cDNA) because NMD is markedly dependent on RNA splicing [24,45]. To achieve a more translational impact, we included pharmacological agents that are already used for the treatment of CF patients (VX-809, VX-445, VX-770) or that are tested in clinical trials (ELX-02, PTI-428). In particular, ELX-02, which is a promising non-toxic alternative to G418 [23,46], was not tested in PTCs other than G542X using a CFTR functional assay [22,46]. Additionally, VX-445, which is a highly effective second-generation corrector, has not been tested before on CFTR PTCs. For NMD suppression, we had to rely on SMG1i, which is a useful research tool but not suitable for in vivo studies. To assess the importance of PTC position, we studied various PTCs localized in regions of the gene corresponding to various domains of CFTR protein: Y122X in TMD1, G542X and R553X in NBD1, R1162X in the TMD2-NBD2 linker region, and W1282X in NBD2.

Regarding readthrough agents, we found a significant effect of G418 and ELX-02, with various degrees of efficacy, on Y122X, G542X, R553X, and R1162X. The striking exception is represented by W1282X, which was totally insensitive to readthrough treatment. For some of the mutations responding to readthrough agents, particularly G542X, corrector VX-809 appeared to improve the rescue, probably because of enhanced stability of the full-length CFTR protein that may have contained an amino acid substitution. In this respect, we tested VX-809 on G542C-CFTR, which is the most probable outcome of G542X readthrough, followed by G542W and G542R [33]. We found that, in agreement with previous results [33], VX-809 significantly enhances G542C-CFTR protein expression. This finding is a probable explanation of the efficacy of G418 plus VX-809 on G542X-CFTR. Intriguingly, VX-445, which is very active as corrector on a variety of CFTR mutants [14], was ineffective on G542X-CFTR rescued by readthrough and on G542C-CFTR. Since VX-445 possibly acts on NBD1 [14], it is tempting to speculate that G542 residue has some role in the VX-445 mechanism of action.

Regarding NMD suppression, we found that SMG1i did not enhance the function of CFTR of G542X, R553X, and R1162X mutations, despite a more than 10-fold increase in CFTR mRNA levels by this compound. This large increase in mRNA levels is expected to provide more substrate for readthrough agents and hence synthesis of full length CFTR. Instead, we found no evidence of additive effects by combining readthrough agents and SGM1i. These findings are different from those obtained in a mouse model of G542X-CFTR [44]. In this study, the intestinal organoid swelling assay, which is a readout of CFTR function, revealed synergy between G418 and SMG1i. The discrepancy with respect to our results could be due to the different cell background and assays. It is also important to remark that previous studies have found that suppression of NMD has a negative impact on readthrough, since UPF1 and other related proteins are required for both processes [47].

Interestingly, our experiments revealed that Y122X shows some sensitivity to the combination of readthrough and NMD suppression. Indeed, SMG1i plus ELX-02 caused a modest but significant increase in Y122X-CFTR activity with respect to ELX-02 alone. This finding may seem surprising since in our assays of CFTR mRNA, we found that Y122X was less responsive to SMG1i (4-fold increase). However, it is important to note that Y122X is less severely affected by NMD than the other nonsense mutations [41]. This behavior would explain the smaller relative efficacy of the NMD inhibitor. It is also interesting to note that the protein truncating effect of Y122X can also be circumvented by alternative translation start sites occurring downstream the mutation [41]. Therefore, despite its localization at the beginning of the CFTR coding sequence, various factors contribute to decrease the severity of this mutation.

In contrast with all other mutations, SMG1i was markedly effective on W1282X at the mRNA, protein, and functional level (although rescue of function for this mutant absolutely required the treatment with VX-445). In theory, we would expect that NMD suppression prevents mRNA degradation, thus providing more substrate for readthrough and hence more full-length protein synthesis. However, we never found evidence of cooperative activity by combining readthrough agents and NMD inhibitor. Such results are similar to those of a previous study, in which inclusion of G418 was even detrimental to rescue by SMG1i [42]. It is known that the NMD process is triggered when a ribosome is stalled at a PTC, preventing the displacement of the remaining exon-junction complexes [24]. Therefore, continuation of protein synthesis by readthrough should result in total exon junction complex displacement and prevention of NMD. However, this effect was not seen in our experiments: G418 treatment did not result in enhanced mRNA levels (Figure 8B). The marked effect of SMG1i at the mRNA level corresponded to enhanced function only for W1282X, in which the synthesized protein can be rescued with correctors without the need of readthrough. Further studies are needed to understand why NMD suppression and readthrough do not cooperate. As already cited above, NMD and readthrough share key proteins, so that suppression of NMD may interfere with readthrough [47]. Furthermore, it has been shown that mRNA carrying PTCs are targeted for NMD by transport to specialized regions of cells [47]. It can be speculated that NMD suppression salvages mRNA from degradation but then the resulting mRNA is in a condition/localization that is not sensitive to readthrough. According to this hypothesis, only W1282X-CFTR would be rescuable at the functional level since it may work as a Cl^−^ channel without the need of readthrough. Intriguingly, immunofluorescence revealed a plasma membrane localization of W1282X-CFTR with just SMG1i treatment, in the absence of correctors. This implies that the protein, once adequately synthesized in larger amounts by overcoming NMD, is able to traffic to the cell surface. In this respect, it was previously shown that W1282X-CFTR is normally glycosylated [48]. Despite the apparent normal processing and trafficking, our experiments showed that corrector VX-445 is absolutely required to restore function. This behavior suggests a possible effect of this compound on the normalization of CFTR channel gating, as recently proposed [15,16]. However, our experiments indicated that VX-445 does not behave as a classical potentiator, as previously demonstrated for class 2 and class 3 mutations [15,16]. Indeed, acute application of VX-445 did not result in enhanced anion transport. Therefore, improvement in channel gating may require long-term incubation.

Interestingly, we succeeded in generating cells co-expressing W1282X-CFTR and HS-YFP. The functional data obtained with the HS-YFP assay were perfectly in agreement with transepithelial current recordings. In particular, the assay confirmed the particular sensitivity of this mutant to VX-445. The suitability of HS-YFP for high-throughput screenings will allow identification of novel inhibitors of NMD by looking into a large collection of chemical compounds.

The panel of compounds that was tested in our study included PTI-428, a CFTR amplifier that is potentially able to enhance the synthesis of CFTR protein, irrespective of the presence of mutations [31,32]. This compound failed to increase the rescue of G542X-CFTR or W1282X-CFTR. We checked PTI-428 in CFBE41o- cells expressing CFTR with the F508del mutation. As expected, the amplifier was effective by itself and further improved the rescue by VX-809 in a synergistic fashion. PTI-428 was also effective in increasing CFTR function in primary bronchial epithelial cells. CFTR amplifiers act through PCBP1, a protein that interacts with CFTR mRNA [32]. It is possible that, unlike other CF mutations, PTCs interfere with the function of PCBP1. Our experiments on primary bronchial epithelial cells revealed that PTI-428 also significantly enhances the activity associated with ENaC and TMEM16A channels. Such results suggest that CFTR amplifiers may alter the expression/function of other proteins involved in transepithelial ion transport. These effects need further investigation.

Summarizing, we obtained several important indications that may guide a precision medicine approach for treating patients with PTCs. As also indicated by previous studies [42,43], W1282X appears as the most promising mutation to treat, given the large extent of rescue obtained at the functional level. This result requires a combination of NMD suppression and CFTR correction, without inclusion of a readthrough agent that could even exert detrimental effects [42]. We even found in 16HBE14o- cells a small but significant response to correctors in the absence of SMG1i, thus suggesting the presence of a certain level of mRNA escape from NMD. Our study indicates that, in contrast with the other mutations, VX-445 is the appropriate corrector to include since in its absence there is no effect at the functional level. Therefore, treatments including this agent, as the triple drug Trikafta combination that consists of VX-445 plus VX-661 and VX-770, are expected to be beneficial, also taking into consideration the potentiation effect achieved with VX-770. However, other inhibitors of NMD are needed since SMG1i is expected to generate undesired side effects in vivo [43]. In this respect, it is believed that NMD factors cannot be completely shut down since they play an important general role in gene expression regulation [49]. A possible strategy is to modulate regulatory steps of NMD that, instead of totally blocking this pathway, induce a partial inhibition that is tolerated by the organism but is still effective in rescuing CFTR mRNA from degradation. It has also to be considered that the extent of NMD shows inter-individual variability [50]. Therefore, patients with a lower level of NMD could be more sensitive to CFTR correction/potentiation, with a reduced requirement of NMD inhibitors.

As for the other mutations, our experiments show that the rescue of Y122X and G542X with readthrough agents, with the latter mutation being also sensitive to VX-809, brings CFTR function close to 10% of normal level in 16HBE14o- cells. We also found significant rescue (~15% of normal function) in native bronchial epithelial cells with a single G542X allele. These are useful indications for the design of clinical trials in which a safe compound such as ELX-02 can be combined with existing CFTR modulators to test the possibility of obtaining a milder CF condition.

## 4. Materials and Methods

### 4.1. Chemicals

SMG1 inhibitor (SMG1i) was provided by the Cystic Fibrosis Foundation (CFF) and dissolved in dimethyl sulfoxide. ELX-02 disulfate (HY-114231B) was purchased from MedChemExpress (Monmouth Junction, NJ, USA). We weighed the precise quantity of ELX-02 powder for every experiment and dissolved it directly in the culture medium to reach a final concentration of 0.5 mg/mL. G418 Sulphate Powder (Euroclone, Pero, Italy) was dissolved in Dulbecco’s phosphate-buffered saline (Euroclone) at a concentration of 50 mg/mL, filtered and stored at +4 °C. For cell treatment, G418 stock was diluted in the medium to a final concentration of 0.5 mg/mL. VX-809 (Lumacaftor), VX-445 (Elexacaftor), and PTI-428 were purchased from MedChemExpress. VX-770 (Ivacaftor) and CFTR_inh_-172 were purchased from Selleckchem (Houston, TX, USA). All of these compounds were dissolved in DMSO at a concentration of 10 mM. All other chemicals were obtained from Sigma-Aldrich (St. Louis, MO, USA).

### 4.2. Cell Culture

16HBE14o- immortalized bronchial epithelial cells gene-edited to express different PTCs [34] were obtained from the Cystic Fibrosis Foundation (CFF). Cells were cultured at 37 °C in 5% CO_2_ in minimal essential medium (MEM, Euroclone) supplemented with 10% fetal bovine serum (Euroclone), 2 mM glutamine, 100 U/mL penicillin, 100 µg/mL streptomycin. All flasks and plates were previously coated with a solution consisting of LHC-8 basal medium, 1.34 µL/mL of bovine serum albumin (7.5%), and 10 µL/mL bovine collagen solution Type I (Advanced BioMatrix, Carlsbad, CA, USA). The coating solution was left for at least 2–3 h at 37 °C and then removed. Coated flasks and plates were stored at 4 °C until use. For 16HBE14o- freezing and sub-culturing, we followed the instructions provided by CFF.

Generation and culture of CFBE41o- bronchial epithelial cells expressing F508del-CFTR and the halide-sensitive yellow fluorescent protein (HS-YFP) was previously described [37].

The procedures for isolation and culture of human bronchial epithelial cells (HBECs) were described in detail in previous studies [51,52,53]. The collection and study of airway epithelial cells were specifically approved by the Ethics Committee of the Istituto Giannina Gaslini following the guidelines of the Italian Ministry of Health (registration numbers: ANTECER, 042-09/07/2018 and 28/2020). Bronchial epithelial cells from non-CF individuals and G542X/1717-1G > A patient were isolated in our laboratory. Bronchial epithelial cells from a W1282X/W1282X patient were kindly provided by the Cystic Fibrosis Foundation Laboratory. Bronchial cells were cultured in a serum-free medium (LHC basal medium/RPMI 1640, Thermo Fisher Scientific, Waltham, MA, USA) with the addition of a cocktail of various hormones and supplements (for detailed preparation of this medium, see Reference [51]). To further promote proliferation of basal stem cells [54], the medium was supplemented with a BMP antagonist (DMH-1; Tocris, Bristol, UK), a TGF-β antagonist (A 83-01; Tocris), and a ROCK1 inhibitor (Y-27632; Tocris). After four to five passages, cells were seeded at high density (5 × 10^5^/cm^2^) onto Snapwell porous inserts (3801, Corning Costar, New York, NY, USA). After 24 h from seeding, the proliferative medium was switched to differentiation medium (PneumaCult ALI, Stemcell Technologies, Vancouver, BC, Canada) to induce mucociliary differentiation, and cells were directly kept under air–liquid interface for 2–3 weeks.

### 4.3. Short-Circuit Current Recordings

16HBE14o- cells were seeded at high density (5 × 10^5^/cm^2^) onto Snapwell permeable supports, with 12 mm diameter, 0.4 µm pore size (3801, Corning Costar). Cells were grown in submerged cultures for a week by changing the medium daily on both sides of permeable supports. After 6 days, we measured the transepithelial resistance (Rt) to assess formation of electrically tight epithelia. Epithelia with Rt between 400 and 1000 Ω cm^2^ were used for experiments. Twenty-four hours before short-circuit current recordings, cells were treated with compounds or vehicle added in the culture medium on both sides (apical and basolateral) of epithelia. After treatment (day 7 after plating), the Snapwell supports carrying 16HBE14o- epithelia were inserted in P2300 vertical chambers and mounted in an 8-Channel EasyMount Ussing Chamber System (Physiologic Instruments, San Diego, CA, USA). The apical and basolateral hemichambers were filled with solutions of different composition to create a Cl^−^ gradient. The apical chamber was filled with a 5 mL solution containing (in mM): 63 NaCl, 63 sodium gluconate, 0.38 KH_2_PO_4_, 2.13 K_2_HPO_4_, 2 CaCl_2_, 1 MgSO_4_, 20 Na-HEPES, and 10 glucose. The basolateral chamber was instead filled with 5 mL of a solution containing (in mM): 126 NaCl, 0.38 KH_2_PO_4_, 2.13 K_2_HPO_4_, 1 CaCl_2_, 1 MgSO_4_, 20 Na-HEPES, and 10 glucose. Both solutions were adjusted to a pH of 7.4. The apical and the basolateral solutions were continuously bubbled with air, and the temperature of the solution was kept at 37 °C.

For HBECs, symmetrical bicarbonate-buffered solution was used. The composition of this solution was (in mM): 126 NaCl, 0.38 KH_2_PO_4_, 2.13 K_2_HPO_4_, 1 MgSO_4_, 1 CaCl_2_, 24 NaHCO_3_, and 10 glucose. Both sides were continuously bubbled with a gas mixture containing 5% CO_2_/95% air, and the temperature of the solution was kept at 37 °C.

Transepithelial voltage was short-circuited with a voltage clamp (VCC/MC8, Physiologic Instruments, San Diego, CA, USA) connected to the apical and basolateral chambers via Ag/AgCl electrodes and agar bridges (1 M KCl in 2% agar). The offset between voltage electrodes and the fluid resistance was cancelled before experiments. The short-circuit current was recorded with an Acquire & Analyze U100 (Physiologic Instruments, San Diego, CA, USA) analog-to-digital converter connected to a computer and visualized by the Acquire & Analyze 2.3 software.

### 4.4. RNA Extraction and Quantitative Reverse Transcription PCR (RT-qPCR)

16HBE14o- cells expressing the various mutations were seeded in coated 6-well plates at a density of 400,000 cells/well. Twenty-four hours after seeding, cells were treated with vehicle (DMSO), SMG1i (1 µM), and/or G418 (0.5 mg/mL). After 24 h of treatment, cells were lysed in Trizol (Thermo Fisher Scientific), and total RNA was extracted using the ReliaPrep^TM^ RNA cell Miniprep System (Z6011, Promega, Madison, WI, USA). cDNAs were synthesized using LunaScript^®^ RT SuperMix kit (E3010L, New England BioLabs, Ipswich, MA, USA). Real-time PCR was carried out using TaqMan™ Gene Expression Assays (Thermo Fischer Scientific) for CFTR (Assay ID: Hs00357011_m1) and for B2M (Assay ID: Hs00187842_m1). The real-time PCR reaction was run in a Light Cycler 96 (Roche, Basel, Switzerland). We performed a relative quantitation of CFTR transcript in cells treated with G418 and/or SMG1i compared with control. CFTR expression levels were calculated by subtracting reference gene (B2M) cycle threshold (Ct) values from target gene (CFTR) Ct values to normalize for total input, resulting in ΔCt levels. Relative CFTR transcript abundance was computed as 2^−ΔCt^.

### 4.5. HS-YFP Assay

16HBE14o- cells with W1282X-CFTR, and CFBE41o- cells with F508del-CFTR, both with co-expression of HS-YFP, were plated at high-density in bottom clear, black 96-well microplates (3603, Corning Costar). After 24 h, cells were indicated with compounds or vehicle. After a further 24 h, cells were studied with the HS-YFP assay for the determination of CFTR activity, as previously described [37,38]. Briefly, culture medium was removed by washing three times with complete PBS. Then cells in each well were incubated for 20–25 min with 60 µL PBS supplemented with forskolin (20 µM) plus VX-770 (1 µM) to maximally stimulate CFTR activity. The microplates were then transferred to a microplate reader (FLUOstar Omega, BMG Labtech, Offenburg, Germany) for CFTR activity determination. The plate reader was equipped with high-quality excitation and emission filters for YFP (ET500/20x and ET535/30m, respectively) (Chroma Technology, Rockingham, VT, USA). HS-YFP fluorescence was recorded, with 0.2 s sampling time, for 2 s before and 12 s after injection of 165 µL of an iodide-containing solution (PBS with Cl^−^ replaced by I^−^; final I^−^ concentration: 100 mM). Data were normalized to the initial background-subtracted fluorescence. To determine the fluorescence quenching rate (QR), caused by iodide influx through CFTR, the final 11 s of the data for each well were fitted with an exponential function to extrapolate initial slope (dF/dt).

### 4.6. Western Blot

Two days before the experiment, 16HBE14o- cells carrying different CFTR mutations were seeded at a concentration of 400,000 cells per well on 6-well culture plates (Corning Costar) and then treated with vehicle or indicated compounds the day after. HEK293T cells were also grown on 6-well culture plates until 60% of confluence and then transfected the day prior the experiment with 2 µg total plasmid DNA. After 6 h from transfection, medium was changed, and cells were treated with vehicle or the indicated treatment combinations. Cells where lysed in radioimmune precipitation assay buffer (50 mM Tris-HCl pH 7.4, 150 mM NaCl, 1% Triton X-100, 0.5% sodium deoxycholate, 0.1% SDS) containing Complete Protease Inhibitor Cocktail (Roche) and then centrifuged at 14,500× *g* at 4 °C for 15 min. Total protein concentration of the centrifuged supernatant was calculated using the BCA assay (Euroclone), following the manufacturer’s instructions. Equal amounts of protein (20 µg to detect CFTR and GAPDH for HEK293T transfected cells and 40 µg to detect CFTR and GAPDH in 16HBE14o- cells) were then separated onto gradient (4–15%) Criterion TGX Precast gels (Bio-Rad, Hercules, CA, USA), transferred to nitrocellulose membrane with Trans-Blot Turbo system (Bio-Rad) and analyzed by Western blotting. CFTR protein was detected with mouse monoclonal anti-CFTR antibody (596, kindly provided by Cystic Fibrosis Foundation and University of North Carolina, Chapel Hill) diluted 1:2500; GAPDH was instead immunodetected with anti-GAPDH antibody MAB374 (Merck-Millipore, Darmstadt, Germany) diluted 1:5000. Both were followed by polyclonal anti-mouse HR-conjugated secondary antibody diluted 1:10000 (P0447, Dako, Agilent, Santa Clara, CA, USA) and subsequently revealed by chemiluminescence using the SuperSignal West Femto Substrate (Thermo Fisher Scientific). Direct imaging of the chemiluminescence was obtained using the Molecular Imager UVITEC Cambridge System. Images were analyzed with Fiji software (National Institutes of Health, Bethesda, MD, USA). Total band C was analyzed as a region of interest normalized against the GAPDH control.

### 4.7. Immunofluorescence

The 16HBE14o- cells expressing G542X- and W1282X-CFTR were seeded on µ-Slide 12-well removable chamber support (Ibidi, Gräfelfing, Germany), previously coated with LHC8 coating medium, at a confluency of 20,000 cells per well in a total volume of 200 µL of complete MEM medium (supplemented with 10% FBS; 1% penicillin–streptomycin; 1% glutamine). Immunostaining was performed as previously described [13]. Briefly, the day before the experiment, cells were treated with vehicle and the indicated treatment combinations. After 24 h, cells were fixed with 10% neutral buffered formalin (0501005Q, Bio-Optica, Milan, Italy) for 5 min at room temperature. After three washings in PBS, cells were permeabilized and blocked by adding a blocking buffer containing saponin as a permeabilizing agent and BSA (1X PBS; 0.5% BSA; 50 mM NH_4_Cl, 0.02% NaNH_3_, 0.05% saponin) for 30 min at room temperature. After blocking, cells were incubated overnight at 4 °C with primary antibody diluted in the blocking buffer. Rabbit IgG anti-CFTR (D6W6L, Cell Signaling Technology, Danvers, MA, USA) at 1:400 was used as primary antibody.

Following incubation with primary antibody, cells were incubated with Alexa Fluor 488-conjugated secondary antibody (Thermo Fisher Scientific) diluted 1:200 in blocking buffer for 1 h in the dark. After 3 washes in PBS, the silicone chamber was removed, and cells were mounted with Fluoroshield with DAPI (Sigma-Aldrich) to stain cell nuclei. Image acquisition was performed using a laser scanning confocal microscope (Zeiss LSM 700, Oberkochen, Germany). Image analysis was performed using Leica and ImageJ (NIH) software.

### 4.8. Data Presentation and Statistics

Data are presented as representative traces/images and/or as scatter dot plots (plus mean and standard deviation). Statistical significance was determined with Prism 9.1.0 software (GraphPad Software Inc., La Jolla, CA, USA) using, in cases of more than two groups of data, ANOVA followed by Tukey’s post hoc test. For real-time PCR data, we evaluated the CFTR transcript fold-change using one-sample *t*-tests and considering the CFTR expression value in the control condition as 1.

## Data Availability

The data presented in this study are available in the article and Supplementary Materials.

## Supplementary Materials

The following are available online at https://www.mdpi.com/article/10.3390/ijms222111972/s1.
