# Supplementary material for: Comprehensive Analysis of Combinatorial Pharmacological Treatments to Correct Nonsense Mutations in the CFTR Gene
Source: Int J Mol Sci. 2021 Nov 4;22(21):11972. doi: 10.3390/ijms222111972 (PMC8584557; doi:10.3390/ijms222111972)
Supplement: Supplementary file 1 [file ijms-22-11972-s001.zip › ijms-1313691-supplementary.pdf]

Venturini et al.

SUPPLEMENTARY FIGURES

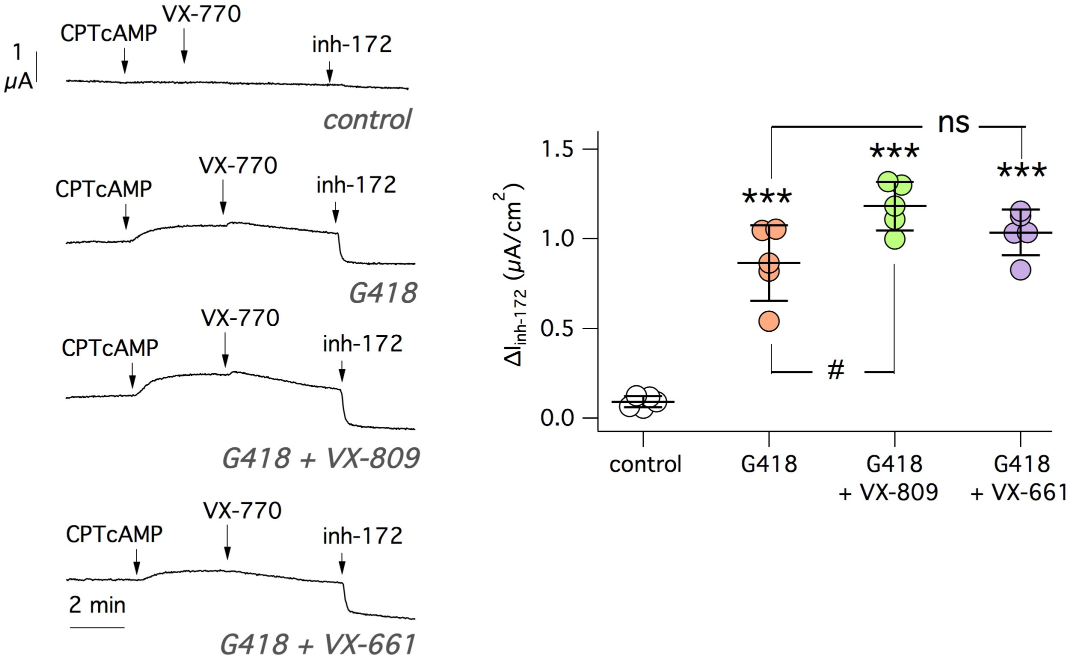

Figure S1. Analysis of G542X-CFTR rescue by VX-809 or VX-661. Representative traces (left) and summary of data (right) from short-circuit current experiments done on 16HBE14o- cells expressing G542X-CFTR. Cells were treated for 24 hours with G418 (0.5 mg/ml), with/without VX-809 (1 µM) or VX-661 (5 µM). ***, $p < 0.001$ vs. control; #, $p < 0.05$ vs. G418; ns, not significant.

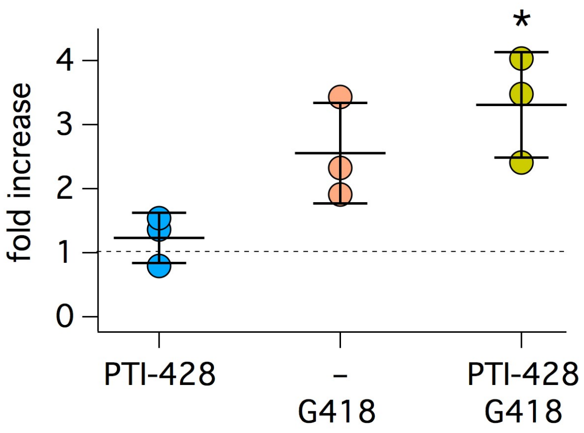

Figure S2. Quantitative analysis of CFTR mRNA. 16HBE14o- cells expressing G542X-CFTR were treated for 24 hours with PTI-428 (30 µM), G418 (0.5 mg/ml) or both compounds. After treatment, RNA was extracted, converted to cDNA, and CFTR transcript quantified by real time RT-PCR. Results in each sample were normalized to B2M expression. The scatter dot-plot shows CFTR transcripts fold-change relative to control condition. *, $p < 0.05$ vs. vehicle treated cells (dashed line).

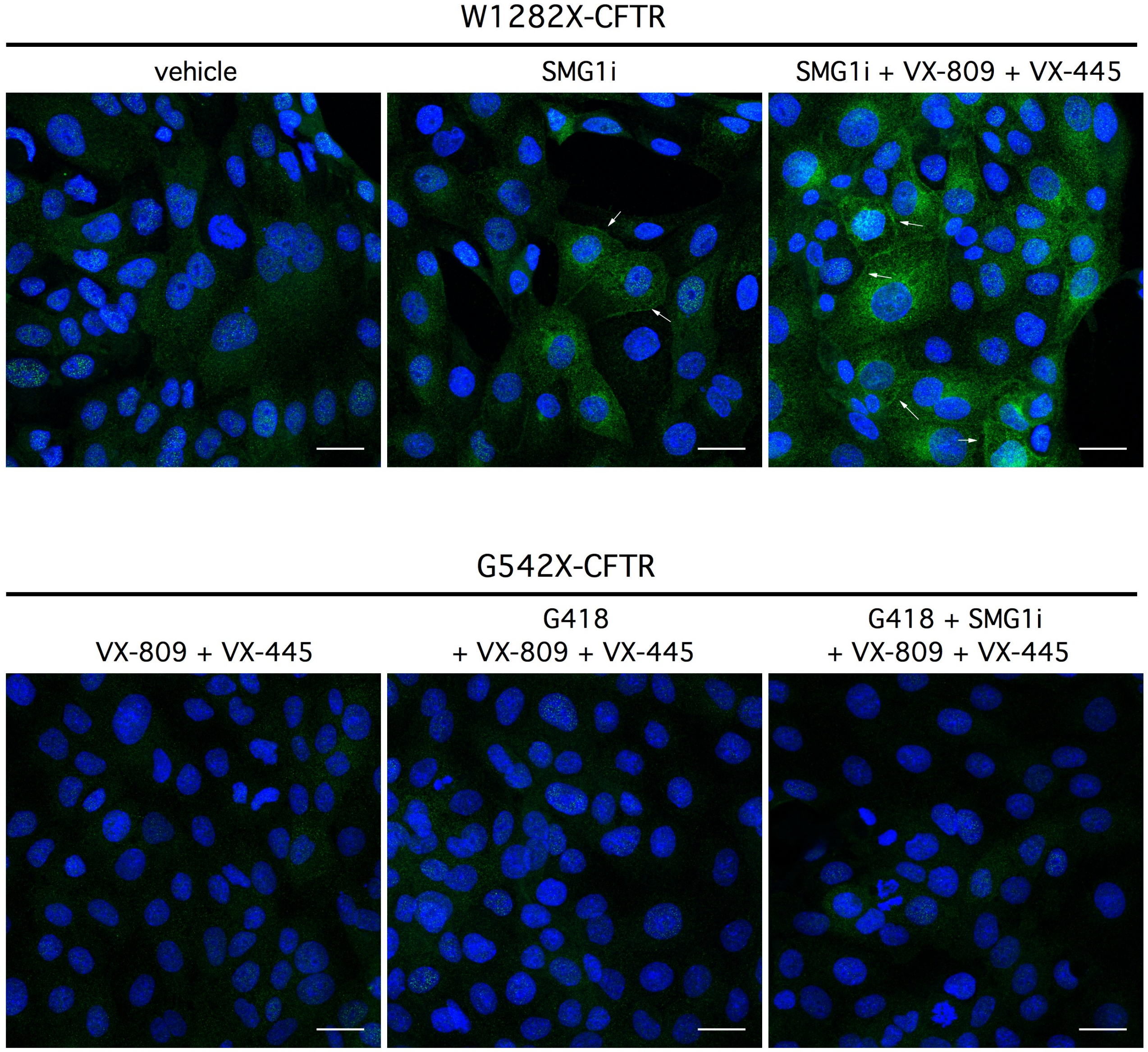

Figure S3. Immunofluorescence analysis of CFTR rescue. Representative images from W1282X- and G542X-CFTR cells treated with SMG1i (1 µM) alone or in combination with VX-809 (1 µM) plus VX-445 (5 µM). Cells expressing the W1282X mutant show appearance of immunoreactive CFTR (green) following pharmacological treatment. Arrows: appearance of CFTR signal at cell borders. Scale bar: 20 µm.

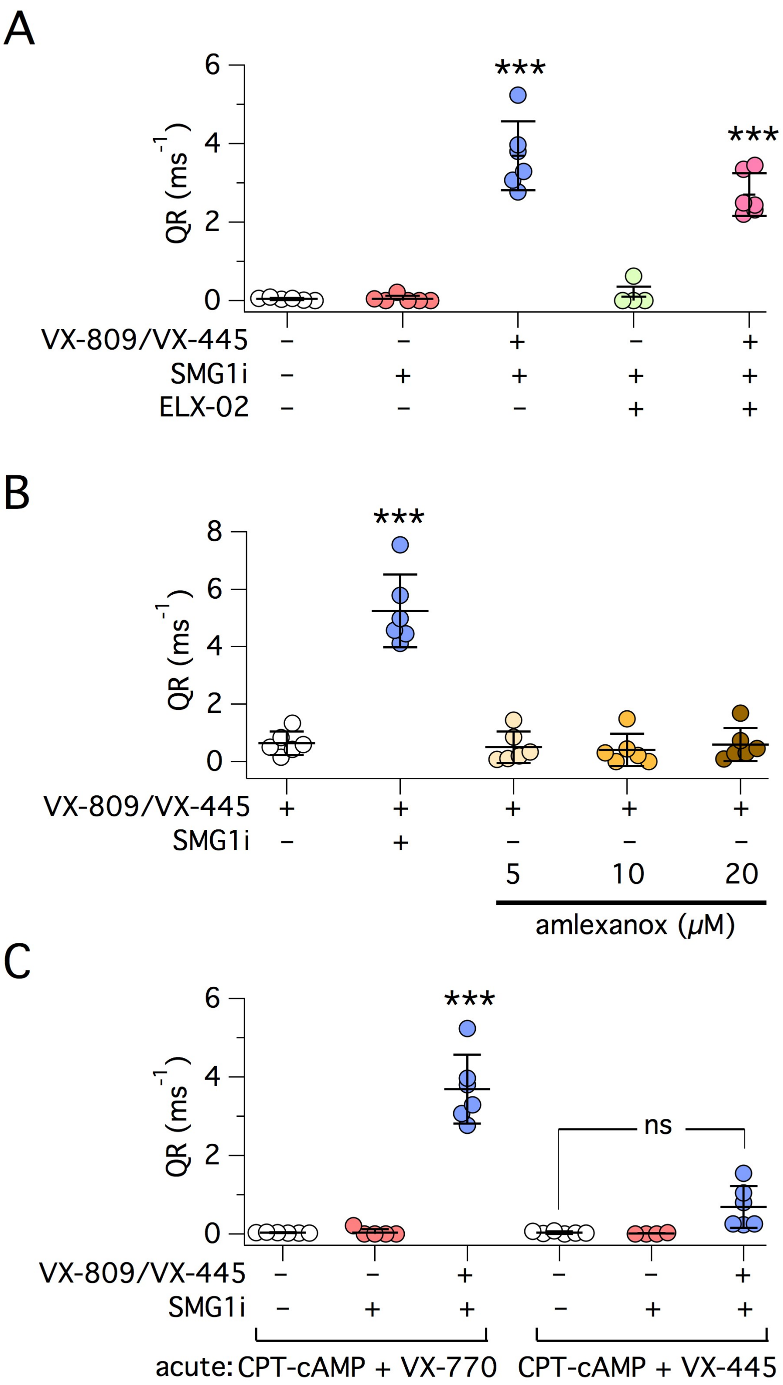

Figure S4. Evaluation of W1282X-CFTR function with the HS-YFP. (A) Evaluation of W1282X-CFTR rescue by SMG1i alone or in combination with ELX-02 with/without VX-809 (1 μM) and VX-445 (5μM). After treatment, W1282X-CFTR activity was determined with the HS-YFP assay. After treatment, W1282X-CFTR activity was determined with the HS-YFP assay. ***, $p < 0.001$ vs. control. (B) Evaluation of amlexanox as a NMD inhibitor. Cells were treated for 24 hrs with VX-809 (1 μM) plus VX-445 (5 μM), with/without amlexanox (5-10-20 μM) or SMG1i (1 μM). In contrast to SMG1i, amlexanox was ineffective. ***, $p < 0.001$ vs. correctors alone. (C) Evaluation of VX-445 as a CFTR potentiator. Cells were treated for 24 hours with vehicle or SMG1i (1 μM) plus/minus VX-809 (1 μM) and VX-445 (5 μM). After treatment, cells were acutely stimulated with CPT-cAMP (100 μM) plus VX-770 (1 μM) or VX-445 (5 μM). ***, $p < 0.001$ vs. vehicle.
